# Postprandial Hyperlipidemia: Its Pathophysiology, Diagnosis, Atherogenesis, and Treatments

**DOI:** 10.3390/ijms241813942

**Published:** 2023-09-11

**Authors:** Hidekatsu Yanai, Hiroki Adachi, Mariko Hakoshima, Hisayuki Katsuyama

**Affiliations:** Department of Diabetes, Endocrinology and Metabolism, National Center for Global Health and Medicine, Kohnodai Hospital, 1-7-1 Kohnodai, Ichikawa 272-8516, Chiba, Japan; dadachidm@hospk.ncgm.go.jp (H.A.); d-hakoshima@hospk.ncgm.go.jp (M.H.); d-katsuyama@hospk.ncgm.go.jp (H.K.)

**Keywords:** apo B48, cardiovascular disease, insulin resistance, postprandial hyperlipidemia, remnant cholesterol, small dense LDL

## Abstract

Postprandial hyperlipidemia showing postprandial increases in serum triglyceride (TG) is associated with the development of atherosclerotic cardiovascular disease (ASCVD). To diagnose postprandial hyperlipidemia, the oral fat loading test (OFLT) should be performed; however, this test is very time-consuming and is difficult to perform. Elevated serum TG levels reflect an increase in TG-rich lipoproteins (TRLs), such as chylomicrons (CM), very low-density lipoproteins (VLDL), and their remnants (CM remnants [CMRs] and VLDL remnants [VLDLRs]). Understanding of elevation in CMR and/or VLDLR can lead us to understand the existence of postprandial hyperlipidemia. The measurement of apo B48, which is a constituent of CM and CMR; non-fasting TG, which includes TG content in all lipoproteins including CM and CMR; non-high-density lipoprotein cholesterol (non-HDL-C), which includes TRLs and low-density lipoprotein; and remnant cholesterol are useful to reveal the existence of postprandial hyperlipidemia. Postprandial hyperlipidemia is observed in patients with familial type III hyperlipoproteinemia, familial combined hyperlipidemia, chronic kidney disease, metabolic syndrome and type 2 diabetes. Postprandial hyperlipidemia is closely related to postprandial hyperglycemia, and insulin resistance may be an inducing and enhancing factor for both postprandial hyperlipidemia and postprandial hyperglycemia. Remnant lipoproteins and metabolic disorders associated with postprandial hyperlipidemia have various atherogenic properties such as induction of inflammation and endothelial dysfunction. A healthy diet, calorie restriction, weight loss, and exercise positively impact postprandial hyperlipidemia. Anti-hyperlipidemic drugs such pemafibrate, fenofibrate, bezafibrate, ezetimibe, and eicosapentaenoic acid have been shown to improve postprandial hyperlipidemia. Anti-diabetic drugs including metformin, alpha-glucosidase inhibitors, pioglitazone, dipeptidyl-peptidase-4 inhibitors and glucagon-like peptide 1 analogues have been shown to ameliorate postprandial hyperlipidemia. Although sodium glucose cotransporter-2 inhibitors have not been proven to reduce postprandial hyperlipidemia, they reduced fasting apo B48 and remnant lipoprotein cholesterol. In conclusion, it is important to appropriately understand the existence of postprandial hyperlipidemia and to connect it to optimal treatments. However, there are some problems with the diagnosis for postprandial hyperlipidemia. Postprandial hyperlipidemia cannot be specifically defined by measures such as TG levels 2 h after a meal. To study interventions for postprandial hyperlipidemia with the outcome of preventing the onset of ASCVD, it is necessary to define postprandial hyperlipidemia using reference values such as IGT.

## 1. Introduction

Postprandial serum triglyceride (TG) fluctuates greatly depending on the content of the meal and the time elapsed after the meal. Therefore, postprandial blood sampling results in higher TG levels than in the fasting state, making it difficult to diagnose dyslipidemia. For this reason, fasting TG values have been evaluated as the definition of dyslipidemia for a long time. Since Zilversmit pointed out the relationship between postprandial hyperlipidemia and atherosclerosis in 1979, and attention has been paid to postprandial hyperlipidemia [1]. Several studies that compared patients who had coronary artery disease (CAD) with controls have demonstrated a significant elevation in postprandial TG after an oral fat loading test (OFLT) in CAD patients [2], and that an increase in postprandial TG is an independent predictor of CAD [3]. To diagnose postprandial hyperlipidemia, an OFLT should be performed; however, there is no consensus about the time to start the postprandial test: the morning, the afternoon, or at night [3,4,5,6]. This test is very time-consuming, and is difficult to perform. In healthy participants, serum levels of TG reach a peak at 3–4 h after a meal, and slowly return to initial serum levels 6–8 h after a meal [7]; therefore, bed rest for over 6 h is required [8]. The energy and fat load are high in the OFLT, making the study of postprandial hyperlipidemia using the OFLT a very non-physiologic affair. In addition, such energy and fat overloads cause nausea, vomiting, and diarrhea in subjects. Therefore, a method to appropriately evaluate postprandial hyperlipidemia without using the OFLT id desirable.

Serum TG levels are considered to be the surrogate marker for TG-rich lipoproteins (TRL) such as chylomicrons (CM), very low-density lipoproteins (VLDL), and their remnants. Significant increases in postprandial TRL are known to predict the risk of CAD [9,10], independent of elevated low-density lipoprotein cholesterol (LDL-C) or reduced high-density lipoprotein cholesterol (HDL-C). 

Zilversmit first proposed that postprandial CM is the most common risk factor for atherosclerotic cardiovascular disease (ASCVD) in persons without familial hyperlipoproteinemia [1]. CM and CM remnants (CMRs) were considered to be the major lipoproteins in postprandial hyperlipidemia until recently. Studies using an immune separation method which enables the direct isolation of remnant lipoproteins as remnant-like lipoprotein particles (RLP) have demonstrated that the major remnant lipoproteins in postprandial hyperlipidemia are not CMR, but VLDL remnants (VLDLRs) [11,12].

CMRs and VLDLRs have multiple atherogenic properties, and increased RLP–cholesterol (RLP-C) concentrations are associated with increased all-cause mortality in patients with ischemic heart disease and the development of CAD, after adjusting for other major risk factors [13].

Here, we discuss the diagnosis, causative diseases, mechanisms of development, and atherogenic properties of, and therapeutic approaches to postprandial hyperlipidemia.

## 2. Evaluation of Postprandial Hyperlipidemia

### 2.1. OFLT

Masuda, D. et al. studied the effect of ezetimibe, which inhibits the Niemann–Pick C1-Like 1 (NPC1L1) protein, an intestinal cholesterol transporter, on postprandial hyperlipidemia in patients with type IIb hyperlipidemia, by performing an OFLT [14]. After an overnight fast for 12 h, OFLT cream containing 35% fat without sugar was administered to each patient, and was sufficient to provide a fat level of 30 g fat/m^2^. Blood samples were drawn before and 1, 2, 3, 4, 6, and 8 h after OFL, and serum levels of total cholesterol (TC), TG, apo B48, free fatty acid (FFA), remnant lipoprotein cholesterol (RemL-C), and apo B100 were measured. 

### 2.2. The Contribution of Remnant Lipoproteins to Postprandial Hyperlipidemia

In the study by Masuda, D. et al., serum TG levels reached a peak at 3–4 h after OFL and slowly returned to initial levels at 6–8 h after OFL in patients with type IIb hyperlipidemia, when they did not take ezetimibe [14]. In this study and the previous study, apo B48 levels reached a peak at 3–4 h after OFL, and slowly returned to initial levels at 6–8 h after OFL. Since apo B48 is a constituent of CM and CMR, the peak of TG at 3–4 h after OFL may be caused partially by the peak in CM or CMR. RemL-C levels also reached a peak at 3–4 h after OFL, supporting a contribution of CMR to the postprandial TG peak. Measurement of apo B48 is useful for evaluating CMR.

In a study that investigated postprandial hyperlipidemia in patients with type 2 diabetes, serum levels of VLDL-TG and VLDL-apo B100 reached a peak at 3–4 h after the ingestion of a test meal, and slowly return to initial levels after 6–8 h [15]. In the study by Nakajima, K. et al. (2012), remnant-like lipoprotein particles (RLP) and apo B100, but not RLP apoB48, increased significantly when the plasma TG increased after OFL, despite the increase in plasma apo B48. The results show that the major lipoproteins increased in postprandial plasma were VLDLR, not CMR [16]. 

Serum TG levels reached a peak at 3–4 h after a meal and slowly returned to initial levels at 6–8 h after a meal in healthy subjects. Serum TG levels are abnormally elevated after a meal, and the peak is delayed or protracted in patients with postprandial hyperlipidemia. An elevation in TG after a meal may be induced by an increase in TRLs such as CM, CMR, VLDL and VLDLR. 

### 2.3. The Evaluation of Remnant Lipoproteins in the Clinical Setting

**Figure 1:**
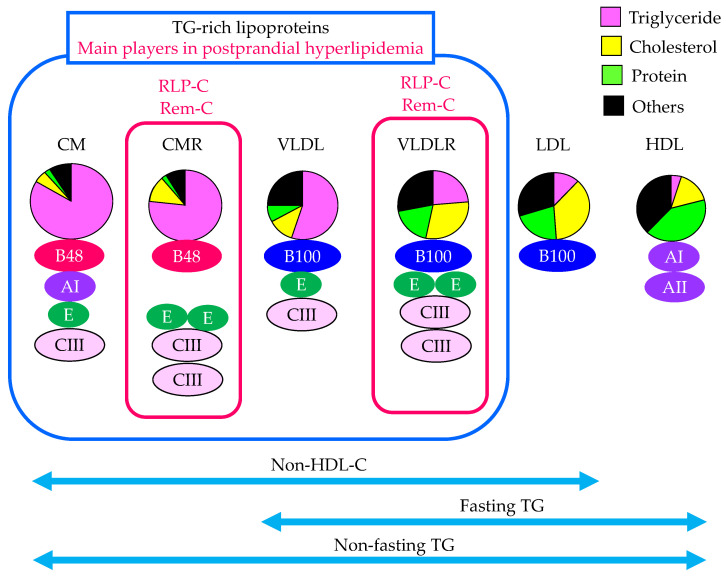
Characteristics of lipoproteins, including TG-rich lipoproteins, which are the main players in postprandial hyperlipidemia, and methods used to evaluate TG-rich lipoproteins. The apoproteins possessed by each lipoprotein exhibited only characteristic apoproteins. CM, chylomicron; CMR, CM remnant; HDL, high-density lipoprotein; Rem-C, remnant lipoprotein cholesterol; RLP-C, remnant-like lipoprotein particle cholesterol; TG, triglyceride; VLDL, very-low-density lipoprotein; VLDLR, VLDL remnant.

The characteristics of lipoproteins including TRLs, which are the main players in postprandial hyperlipidemia, and the methods used to evaluate TRL are shown in Figure 1. TRLs are the main players in postprandial hyperlipidemia. To understand and adequately evaluate postprandial hyperlipidemia, it is important to grasp how changes in TRLs occur. 

#### 2.3.1. Fasting TG

Since serum TG levels vary greatly depending on the content of meals and the postprandial time, serum TG levels in a fasting state have been evaluated for a long time to diagnose dyslipidemia. As seen in Figure 1, the measurement of fasting TG cannot detect elevations in CM and CMR. 

#### 2.3.2. Non-Fasting TG

Non-fasting TG includes the TG content of all lipoproteins including CM and CMR. An epidemiological study in Japan has shown that the risk of developing CAD increases due to elevated non-fasting TG levels, and that a 1 mmol/L (88.6 mg/dL) increase in non-fasting TG increased the CAD risk by 1.29 times in men and 1.42 times in women [17]. In this study, the CAD risk was increased at a non-fasting TG level of 115 mg/dL or higher, and the risk was more than tripled at 167 mg/dL or higher, even after adjusting for HDL-C [17]. In a sub-analysis of the Multiple Risk Factor Intervention Trial (MRFIT), compared with TG levels less than 200 mg/dL, the risk factor-adjusted hazard ratios for coronary heart disease (CHD) mortality for hypertriglyceridemia were 1.24 (*p* = 0.09) for fasting and 1.26 (*p* = 0.07) for non-fasting [18]. For nonfatal or fatal CHD, fasting and non-fasting TG levels were similarly predictive, with hazard ratios of 1.64 (*p* = 0.004) for fasting and 1.46 (*p* = 0.03) for non-fasting. Non-fasting TG levels was suggested to be more useful than fasting measurements for CHD risk stratification because of the greater ease of obtainment, greater prevalence, and similarly increased risk of CHD compared with fasting TG levels. After that, the optimal diagnostic threshold for non-fasting TG values for incident cardiovascular (CV) events was determined by using baseline non-fasting samples from 6391 participants in a Women’s Health Study, who were followed prospectively for 17 years [19]. The optimal threshold of 175 mg/dL (1.98 mmol/L) was found to be statistically better than the American Heart Association (AHA) cut-point of 200 mg/dL [19]. In the United States, a persistent non-fasting serum TG of 175 mg/dL or higher is considered to be a risk-enhancing factor for atherosclerotic CV disease (ASCVD), and in Europe, a non-fasting serum TG of 175 mg/dL or higher is set as the cut-off value for a clinical diagnosis of dyslipidemia [20,21]. Additionally, then, a fasting serum TG of 150 mg/dL or higher and a non-fasting serum TG of 175 mg/dL or higher have been adopted as diagnostic criteria for dyslipidemia in Japan [22]. An increase in non-fasting TG may reflect an elevation in CMR and VLDLR. 

#### 2.3.3. Non-HDL-C

Since non-fasting non-HDL-C is an index that increases with an increase in both LDL-C and TRL, including CMR and VLDLR, when non-HDL-C increases and the LDL-C level is within the normal range, an increase in TRL is suspected.

#### 2.3.4. Fasting Serum Apo B48

The subjects studied were given a high-fat or standard meal, and serum lipid profiles were investigated after each meal [23]. Serum levels of TG, apo B48, RLP-C, and RLP-TG were elevated, and their increases were significantly larger after intake of a high-fat meal as compared with a standard meal. Fasting apo B48 levels were significantly correlated with the incremental area under the curve (iAUC) of TG after intake of the high-fat meal, but not the standard meal. The measurement of fasting serum apo B48 may be an useful method to assess the existence of postprandial hyperlipidemia.

#### 2.3.5. RLP-C

To evaluate remnants such as CMR and VLDLR, remnant cholesterol can be measured using RLP-C and RemL-C methods. Nakajima, K. et al. (1993) have developed a simple, rapid assay method for apo E-rich lipoproteins using an immunoaffinity gel mixture of anti-apo B100 and apo AI antibodies [11]. The immunoaffinity gel mixture adsorbed lipoproteins containing apo AI and apo B100. Unbound lipoprotein cholesterol was quantified. Characterization of the unbound lipoproteins has shown that they represent CM-RLP and VLDL-RLP [24]. Using this method, CM and HDL are bound and removed by a solid phase extracted with apo AI antibody, and nascent VLDL and LDL are bound and removed by a solid phase extracted with apo B100 antibody; RLP is separated into unbound fractions, and their cholesterol concentrations are measured. Since the anti-human apo B100 monoclonal antibody used in this method has an epitope that recognizes the apo B51 position of the apo B100 protein, it does not bind to lipoprotein particles carrying apo B48. Since CM has apo B48 but also has apo AI, CM is not detected in the RLP fraction. CM present in the RLP fraction is considered to be CMR because it does not have apo AI, because CM without apo AI is considered to be CMR [25]. Apo E-rich VLDL, which accounts for about one-third of VLDL containing apo B100, is isolated as RLP because it does not react with the anti-apo B100 antibody used in this method. The RLP-C values measured using this method are the sum of the cholesterol of CMR without apo AI and apo B100, and VLDLR, which has apo B100 and is apo E-rich. RLP is apo CIII-rich as well as apo E-rich, and the idea that apo CIII-rich is also important, because alongside being apo E-rich, it is characteristic of remnant lipoproteins [26]. 

#### 2.3.6. RemL-C

RemL-C utilizes surfactant and phospholipase-D, which selectively solubilize and degrade remnant lipoproteins [27]. Released cholesterol was then measured enzymatically. RemL-C was significantly and positively correlated with RLP-C (R = 0.95, *p* < 0.0001) [28]. Unlike RLP-C, this assay can be run on an automated clinical analyzer, thereby allowing quick and high-throughput measurement. The correlations and data validation between RemL-C and RLP-C were evaluated [29]. A positive and significant correlation was found between the two methods (R = 0.853, *p* < 0.0001). There were several data dissociations between the RemL-C and RLP-C. High-performance liquid chromatography (HPLC) showed high-serum CM-C samples were more strongly associated with RemL-C levels as compared with RLP-C levels, but high-serum IDL-C samples were more significantly associated with RemL-C level as compared with RLP-C level. 

## 3. Pathological Factors That Induce Postprandial Hyperlipidemia

The elevated remnant lipoproteins associated with postprandial hyperlipidemia are also associated with hypertriglyceridemia, low HDL-C levels, and increased small dense LDL (Sd-LDL). Elevated remnant lipoproteins are observed in patients with familial type III hyperlipoproteinemia, familial combined hyperlipidemia (FCHL), chronic kidney disease (CKD), metabolic syndrome, and type 2 diabetes. 

### 3.1. Familial Type III Hyperlipoproteinemia

Familial type III hyperlipoproteinemia is a genetic disorder characterized by an increase in serum remnant lipoproteins and premature development of atherosclerosis [30]. Although receptor binding-defective apo E forms (E2/E2) are the common denominator, other pathogenic factors still exist. Hypercholesterolemia is induced by an impairment of receptor-mediated clearance. Hypertriglyceridemia is induced by impaired lipolysis of remnant lipoproteins and increased VLDL production due to increased apo E levels. Therefore, an elevation in CMR and IDL (VLDLR) is observed in patients with familial type III hyperlipoproteinemia. In such patients, serum TG levels reached a peak at 6–8 h after the OFL, and did not return to initial levels even after 8 h [31], suggesting that familial type III hyperlipoproteinemia causes postprandial hyperlipidemia. 

Familial type III hyperlipoproteinemia is mainly found in homozygous carriers of apo E2. Only a small percentage (<5%) of these apo E2 homozygotes develop hyperlipidemia, indicating that additional environmental and genetic factors contribute to the development of familial type III hyperlipoproteinemia. de Beer, F. et al. demonstrated that compared with normocholesterolemic E2/2 subjects, type III hyperlipoproteinemic patients had a significantly increased body mass index (BMI) and prevalence of hyperinsulinemia, suggesting that the expression of type III hyperlipoproteinemia in E2/2 subjects is associated with insulin resistance [32]. 

### 3.2. FCHL

FCHL was previously reported to be caused by a single gene whose inheritance exhibits autosomal dominant inheritance, and increases both blood cholesterol and TG levels, and FCHL was proposed as primary hyperlipidemia, which is common in patients with a history of myocardial infarction [33]. Type IIb is the basic phenotype of FCHL, but type IIa and IV phenotypes can occur depending on diet and age. The first relative has hyperlipidemia with either type IIb, IIa, or IV, and at least one person, including the subject, has type IIb or type IIa hyperlipidemia. The diagnosis of FCHL requires Apo B/LDL-C > 1.0 or the presence of Sd-LDL. 

FCHL patients showed an abnormal postprandial TG characterized by longer-lasting elevated postprandial TG in plasma and CM [34]. Compared with controls, the 24 h TG levels in plasma, CM and non-CM fractions were higher in FCHL patients. The clearance of CM and CMR, assessed by the AUC for a retinyl palmitate curve, was delayed in FCHL patients compared with controls. Postprandial CMR clearance was studied in six patients with FCHL and seven control subjects using the OFLT. The CMR clearance was delayed in FCHL subjects compared with control subjects [35]. Delayed elimination of atherogenic CMR may contribute to the increased risk of premature atherosclerosis in FCHL. In FCHL patients, postprandial apo B100 remained unchanged in TRL and in remnant fractions, suggesting hypersecretion of VLDL [36]. In FCHL subjects, increased fasting plasma apo B48 levels were observed. Overproduction of VLDL is the major characteristic of subjects with FCHL. The postprandial free fatty acid (FFA) AUC was higher in FCHL subjects than in control subjects [37]. The postprandial increase in ketone bodies was four times higher in FCHL patients. As ketogenesis occurs predominantly in hepatocytes, during the postprandial period, an increased flux of FFA to the liver occurs in patients with FCHL, possibly due to an inadequate incorporation of FFA into TG in adipocytes. Impaired metabolism of postprandial FFA may contribute to hepatic VLDL overproduction in FCHL subjects. Patients with FCHL have 2-fold higher serum RLP-C levels, which may contribute to the increased risk of ASCVD [38].

FCHL accounts for an additional 10–20% of premature CAD. FCHL exhibits features similar to the metabolic syndrome in addition to a disproportionate elevation of apo B levels [39]. Genetic studies have yielded little evidence of single gene determinants of FCHL [40]. DNA sequencing shows that rare large-effect variants in genes such as LDL receptors (LDL-R) and lipoprotein lipases (LPL) are found in some FCHL patients, explaining the elevated LDL-C and TG, respectively. In addition, diet, obesity, fatty liver, and diabetes further modulate the expression of the biochemically defined FCHL phenotype [40]. 

### 3.3. CKD

In CKD and proteinuria patients, loss of apo CII, which is LPL activator, into urine impairs VLDL catabolism [41]. In CKD patients with a reduced glomerular filtration rate (GFR), hepatic VLDL production is not elevated, and VLDL catabolism is impaired. Serum apo CIII, which is LPL inhibitor, is increased, and hepatic lipase (HL) activity is reduced [42,43]. Therefore, serum VLDLR (IDL) levels increase in CKD patients. HDL-C decreases, and serum IDL-C and VLDL-C increase in hemodialysis (HD) patients compared to healthy individuals [44]. 

The OFLT was performed in patients with chronic renal failure (CRF) on HD [45]. Fasting serum TG levels were elevated in CRF patients compared to normal controls. Six patients had type IV hyperlipidemia patterns and two patients showed type IIb hyperlipidemia. The OFLT in all CRF patients showed delayed clearance of serum TG and CM. Serum TG and CM levels in CRF did not decrease by 5 h after the OFL. Several abnormalities in lipoprotein protein and lipids such as enrichment of IDL and TG-rich LDL and VLDL with apo B48 were observed in patients with CRF undergoing peritoneal dialysis or HD [46]. These findings strongly suggest an elevation of CMR and VLDLR in CRF patients.

### 3.4. Metabolic Syndrome and Type 2 Diabetes

#### 3.4.1. The Effects of Insulin Resistance on TRL Metabolism

**Figure 2:**
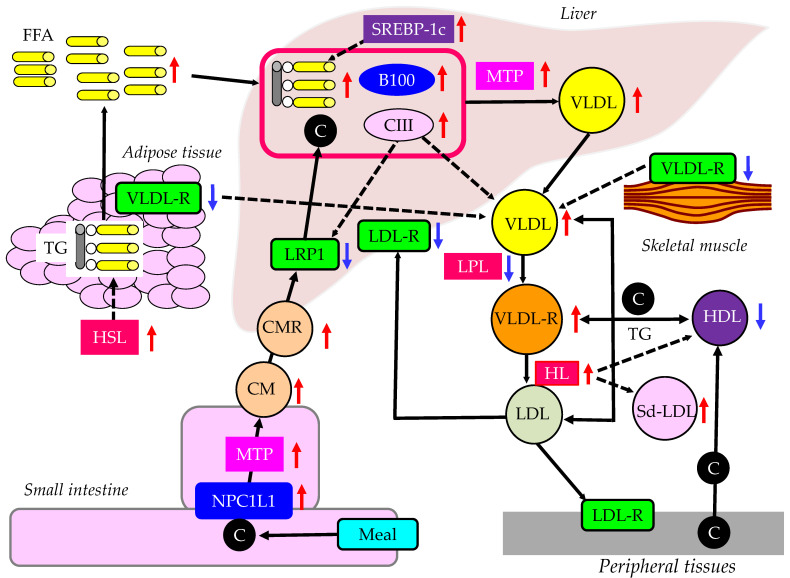
The effects of insulin resistance on TG-rich lipoprotein metabolism. Solid black and dotted lines indicate the flow of substances and effects of metabolic changes, respectively. Red and blue arrows indicate increases and decreases in the activity or expression of metabolic factors, respectively. C, cholesterol; CM, chylomicron; CMR, chylomicron remnant; FFA, free fatty acid; HDL, high-density lipoprotein; HL, hepatic lipase; HSL, hormone sensitive lipase; LDL, low-density lipoprotein; LDL-R, LDL receptor; LRP1, LDL receptor-related protein 1; LPL, lipoprotein lipase; MTP, microsomal triglyceride transfer protein; NPC1L1, Niemann–Pick C1-Like 1; sd-LDL, small dense LDL; SREBP-1c, sterol regulatory element binding protein 1c; TG, triglyceride; VLDL, very-low-density lipoprotein; VLDLR, VLDL remnant; VLDL-R, VLDL receptor.

Metabolic syndrome and type 2 diabetes develop due to insulin resistance. Here, we will consider the effects of insulin resistance on postprandial hyperlipidemia and TRL (Figure 2). Our previous studies showed that the characteristics of dyslipidemia due to insulin resistance are reduced HDL-C and increased IDL-C (VLDLR-C) and VLDL-C, which are further exacerbated by complications associated with obesity [47,48,49,50,51]. The metabolism of FFA is altered in insulin resistance. LPL and hormone-sensitive lipase (HSL) are rate-limiting steps for FFA metabolism in adipose tissue, because LPL hydrolyzes the extracellular TG in lipoproteins, and HSL hydrolyzes the intracellular TG in adipocytes. Relative insulin deficiency due to insulin resistance increases HSL activity and expression in adipose tissue, which catalyzes the breakdown of TG, releasing FFA [52]. Hepatic insulin resistance reduces apo B100 degradation [53]. Insulin resistance increases the expression of microsomal TG transfer protein (MTP), a key enzyme involved in VLDL assembly [54]. In an insulin-resistant state, increased FFA entry to liver, reduced degradation of apoB100, and enhanced expression of MTP may elevate hepatic production of VLDL. Insulin resistance also causes increased expression of sterol regulatory element binding protein 1c (SREBP-1c), which increases FA synthesis [55].

The VLDL receptor binds TRL but not LDL, and functions as a peripheral remnant lipoprotein receptor. The VLDL receptor is expressed abundantly in FA metabolism-active tissues such as the heart, skeletal muscle and adipose tissue. It is likely that VLDL receptor functions in concert with LPL, which hydrolyses TG in VLDL and CM. In contrast to the LDL receptor, gene therapy using the VLDL receptor in the liver showed a beneficial effect on lipoprotein metabolism in both LDL receptor knockout and apo E mutant mice [56]. The VLDL receptor mRNA and protein levels were significantly decreased in skeletal muscle and adipose tissue in hypercholesterolemic, hypertriglyceridemic diabetic rats compared with normal rats [57]. Additionally, in vitro, in 3T3-L1 adipocytes, insulin-induced insulin resistance significantly decreased VLDL-R mRNA expression. 

Insulin resistance also reduces LPL activity. LPL is the rate-limiting enzyme for the catabolism of TRLs such as CM, VLDL and IDL [58]. Therefore, reduced LPL activity decreases the catabolism of TRLs such as VLDL. 

Apo CIII is produced in the liver and small intestine, and has an inhibitory effect on LPL activity. Overexpression of apo CIII in the plasma of transgenic mice results in hypertriglyceridemia, with an up to 20-fold elevation in plasma TG [59]. Total apo B100 levels are similar in transgenic and normal plasma, but apoB48 levels are increased in transgenic mice, which is corrected by the addition of exogenous apo E. The rate of clearance of CMR in apo CIII-transgenic mice was about half that in non-transgenic mice. Lipoprotein alterations are accompanied by up to a 5-fold increase in FFA, which may be the cause of the increased hepatic TG production observed in apo CIII-transgenic mice. Apo CIII modulates the apo E-mediated clearance of TG-rich lipoproteins. 

Overweight individuals with reduced insulin sensitivity often have mild-to-moderate hypertriglyceridemia. Increased hepatic production of VLDL apo CIII is characteristic of subjects with higher body weights and lower levels of insulin sensitivity, and is strongly related to the plasma concentration and level of production of VLDL-TG [60].

A significant and positive correlation was present between plasma HDL-C levels and LPL activity in adipose tissue, suggesting that the activity of LPL in adipose tissue and the rate of catabolism of TRLs might be one of the factors that determine the concentration of HDL in plasma [61]. In conditions with increased atherosclerotic risk, HL activity is often high. HL activity increases with the degree of insulin resistance in type 2 diabetes, and with omental fat mass in women [62,63]. In FCHL and type 2 diabetes, HL may contribute to the development of the atherogenic lipid profile, characterized by low HDL-C levels and the presence of Sd-LDL [62,64]. HL plays a central role in LDL and HDL remodeling. High HL activity is associated with increased Sd-LDL and with reduced HDL-C levels [65]. HL activity is determined by visceral obesity with insulin resistance. Dyslipidemia with high HL activity is a potentially proatherogenic lipoprotein profile in metabolic syndrome, in type 2 diabetes, and in FCHL.

Insulin modulates LDL-R expression and activity. The inactivity of insulin represses LDL-R transcription [66]; however, the association of reduced LDL-R with postprandial hyperlipidemia remains unknown. NPC1L1 plays a pivotal role in intestinal cholesterol absorption. CM and VLDL are abnormally increased in patients with diabetes. The expression of NPC1L1 and MTP was investigated in non-diabetic rats and diabetic cholesterol-fed rats [67]. There was a positive correlation between intestinal NPC1L1 mRNA and CM cholesterol. The diabetic rats had significantly higher CM and VLDL-C, TG, and apo B48 and B100 levels compared with control rats. They had significantly increased NPC1L1 and MTP mRNA in both liver and intestine. Levels of NPC1L1 and MTP mRNA were measured via duodenal biopsies of type 2 diabetic and non-diabetic patients [68]. Diabetic patients had more NPC1L1 mRNA than the control subjects. MTP expression was increased in diabetic patients. There was a positive correlation between NPLC1L1 and MTP mRNA. Increased expression of NPC1L1 and MTP is highly associated with postprandial hyperlipidemia. Postprandial hyperlipidemia is characterized by an increase in CMR. Apo B48 exists in CM and CMR, and fasting apo B48 levels may reflect high postprandial levels of CM and/or CMR. Fasting apo B48 was significantly higher in individuals with metabolic syndrome than those without metabolic syndrome [69]. Another study showed that fasting apo B48 concentration was 40% higher in subjects with metabolic syndrome than those without metabolic syndrome [70]. 

LDL receptor-related protein 1 (LRP1) is an endocytic and signaling receptor expressed in several tissues, and plays a crucial role in clearance of CMR from circulation [71]. LRP1 is involved in insulin signaling, glucose homeostasis in adipocytes, muscle cells, and the brain [71]. Insulin stimulates the translocation of hepatic LRP1 from intracellular vesicles to the plasma membrane, which correlates with an increased uptake of LRP1-specific ligands [72]. In wild-type mice, a glucose-induced insulin response increased the hepatic uptake of LRP1 ligands, while in obese mice with hepatic insulin resistance, insulin-inducible LRP1 ligand uptake was abolished. An impaired hepatic LRP1 translocation can contribute to postprandial hyperlipidemia in insulin resistance. Apo B-containing lipoprotein particles are secreted and cleared by the liver. Insulin plays a key role in the regulation of apo B. Insulin decreases apo B secretion by promoting apo B degradation in the hepatocyte [73]. Insulin also promotes the clearance of circulating apo B particles by the liver via LDL-R and LRP1 [73]. An insulin-resistant state is associated with increased secretion and decreased clearance of apo B. 

#### 3.4.2. Postprandial Hyperlipidemia in Patients with Obesity and/or Type 2 Diabetes

The OFLT demonstrated that patients with type 2 diabetes showed a larger AUC of TG (873.3 ± 527.8 vs. 647.0 ± 218.6 mmol/L), CM-rich TG (CM or CMR) (440.9 ± 317.5 vs. 230.8 ± 125.4), and CM-poor TG (VLDL or VLDLR) (479.5 ± 235.7 vs. 363.7 ± 122.9) than healthy subjects [74]. In both healthy and type 2 diabetic subjects, the total AUC of TG was significantly correlated with fasting TG levels.

The OFLT was performed in 12 type 2 diabetic obese, 15 non-diabetic obese, and 12 non-diabetic non-obese (control) adolescents [75]. The TG AUC was significantly greater in the diabetes group than in the non-diabetic obese and non-diabetic non-obese groups [76]. The postprandial increase in TG was significantly greater in the diabetes group than in the control group. The degree of insulin resistance determined the degree of postprandial hyperlipidemia.

Serum levels of RLP-C and RLP-TG were measured in 541 subjects [76]. Serum RLP-C and RLP-TG were significantly higher in the IGT and diabetic groups compared with the normal group (*p* < 0.001). Further, the incidence of remnant hyperlipoproteinemia in normocholesterolemic subjects was four times higher in the IGT and diabetic groups compared with the normal group.

## 4. Postprandial Hyperglycemia and Postprandial Hyperlipidemia

### 4.1. Abnormal Postprandial Glucose Metabolism in Postprandial Hyperglycemia

**Figure 3:**
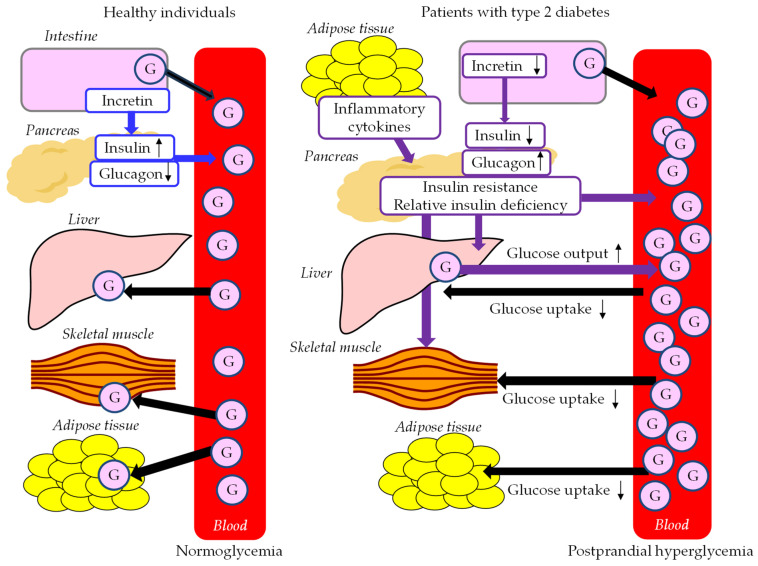
Postprandial glucose metabolism in healthy individuals and patients with type 2 diabetes. Solid black lines indicate the flow of glucose. Solid blue lines indicate the effects of incretin, insulin and glucagon on glucose metabolism in healthy individuals. Solid purple lines indicate the effects of abnormal metabolic factors on glucose metabolism in patients with type 2 diabetes. Black arrows indicate increase (upward) or decrease (downward) of each factor. G, glucose.

Postprandial glucose metabolism in healthy individuals and patients with type 2 diabetes is shown in Figure 3. When a meal is ingested, the plasma glucose level rises first due to the absorption of nutrients. Increased insulin secretion occurs promptly in response to an increase in plasma glucose, and an increase in insulin concentration in the portal vein decreases the endogenous hepatic glucose output (HGO) and increases hepatic glucose uptake (HGU). Increased peripheral plasma glucose due to glucose from the liver causes an increase in glucose uptake by muscle and adipose tissue, resulting in a return of plasma glucose levels to pre-prandial levels. When glucose is taken orally, insulin secretion is increased much more than it is when glucose is intravenously infused, which is called the incretin effect, which accounts for 50 to 70% of the insulin secretion in response to glucose. This incretin effect is induced by glucagon-like peptide-1 (GLP-1) and glucose-dependent insulinotropic polypeptide (GIP) [77]. GLP-1 increases insulin secretion and decreases glucagon secretion. 

Postprandial hyperglycemia in type 2 diabetes is thought to be caused by an increase in splanchnic glucose output (SGO), which is the glucose that enters into the systemic circulation without being taken up by the liver. Insufficient suppression of HGO and decreased HGU are involved in the development of postprandial hyperglycemia in type 2 diabetes [78]. The reasons for the decrease in HGU in type 2 diabetes are that the portal vein insulin concentration does not increase despite the increase in the portal vein glucose concentration; and a sufficient difference in glucose concentration between the postprandial portal vein and the systemic circulation cannot be obtained due to a high pre-prandial plasma glucose level [79]. Hepatic insulin resistance due to fat accumulation may also contribute to the decrease in HGU in type 2 diabetes [79]. Glucose that passes through the liver causes peripheral hyperglycemia; however, it is difficult for it to be taken up by muscle and adipocytes due to decreased insulin action, and postprandial hyperglycemia is thereby prolonged. In patients with type 2 diabetes, the incretin effect is greatly impaired, which induces insufficient insulin secretion and insufficient suppression of glucagon secretion, resulting in postprandial hyperglycemia [77].

### 4.2. Abnormal FFA and TRL Metabolism in Postprandial Hyperlipidemia

**Figure 4:**
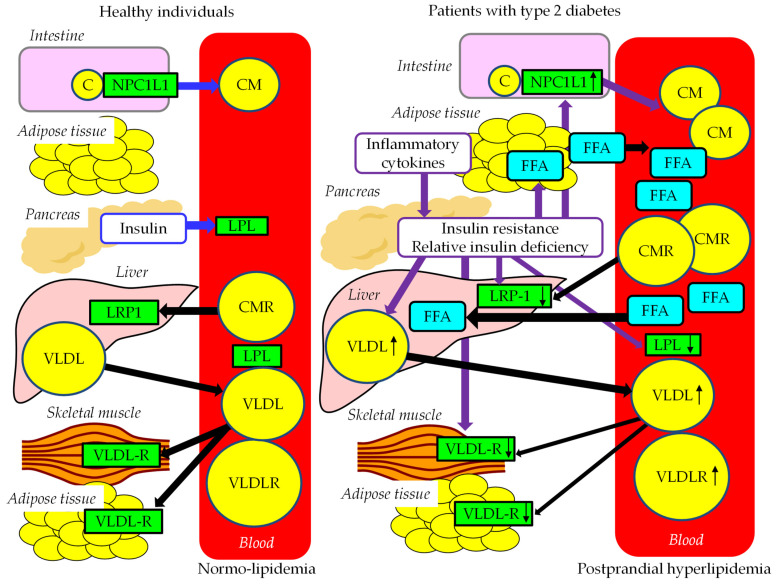
Postprandial FFA and TG-rich lipoprotein metabolism in healthy individuals and patients with type 2 diabetes. Solid black lines indicate the flow of FFA and TG-rich lipoproteins. Solid purple lines indicate the effects of abnormal metabolic factors on FFA and TG-rich lipoprotein metabolism in patients with type 2 diabetes. Black arrows indicate an increase (upward) or decrease (downward) in each factor. C, cholesterol; CM, chylomicron; CMR, chylomicron remnant; FFA, free fatty acid; LRP1, LDL receptor-related protein 1; LPL, lipoprotein lipase; NPC1L1, Niemann–Pick C1-Like 1; VLDL, very-low-density lipoprotein; VLDLR, VLDL remnant; VDLD-R, VLDL receptor.

Postprandial FFA and TRL metabolism in healthy individuals and patients with type 2 diabetes are shown in Figure 4. While glucose acts as an important messenger in postprandial hyperglycemia, FFA acts as an important messenger in postprandial hyperlipidemia. CM is produced by the small intestine. After release into the lymphatic system, secreted CM enters the systemic circulation via the thoracic duct. TG in CM is hydrolyzed by LPL, and TG is hydrolyzed to FFA, which is used in the skeletal muscles as an energy source and/or is stored in adipose tissue in the form of TG. Reduction of TG in CM by LPL makes the CM remnant, and it is then taken up by the liver via LRP1. VLDL is taken up and used by the skeletal muscles and adipose tissue via VLDL-R.

In patients with type 2 diabetes, CM production increases due to increased expression of NPC1L1. The reduced activity of LPL and LRP1 decreases the clearance of CMR. Insulin resistance activates HSL and increases FFA release from adipose tissue, and increased FFA enters into the liver, which increases hepatic VLDL production. The reduced activity of LPL reduces degradation of VLDL. The defective function of VLDL-R in skeletal muscles and adipose tissue further increases serum VLDL. Over production and reduced clearance of CM and VLDL may contribute to the development of postprandial hyperlipidemia.

### 4.3. The Association between Postprandial Hyperglycemia and Postprandial Hyperlipidemia

Postprandial hyperglycemia is commonly observed in patients with metabolic syndrome and type 2 diabetes. Postprandial hyperglycemia is also observed in patients with FCHL [80,81]. However, the postprandial increase in plasma glucose was much larger in patients with type 2 diabetes than in patients with FCHL. The FCHL subjects with BMI ≥ 27 kg/m^2^ showed significantly higher blood glucose and insulin levels following an oral glucose tolerance test than those with BMI < 27 kg/m^2^ [82]. Plasma insulin values were positively related to serum TG. Obesity exacerbates hyperglycemia and hyperinsulinemia in the FCHL subjects. Insulin resistance may enhance postprandial hyperglycemia. 

Young subjects with impaired glucose tolerance/impaired fasting glucose (IGT/IFG) are at higher risk of developing CKD [83]. Children with CKD are at a high risk of glucose intolerance, including postprandial hyperglycemia [84]. Postprandial hyperglycemia and hyperinsulinemia are associated with renal arterio-arteriolosclerosis in CKD [85].

The effects of hyperglycemia and hyperinsulinemia on postprandial RLP concentrations in newly diagnosed type 2 diabetics have been evaluated [86]. Patients with type 2 diabetes were divided on the basis of their plasma insulin response to oral glucose into hyper-insulinemic and normo-insulinemic groups. Serum TG, RLP-TG and RLP-C peaked 2 h after the OFL in the control group, returning to baseline within 4 h. In contrast, such lipids increased throughout the 4 h study in both groups of patients with type 2 diabetes. Increases in RLP-TG and RLP-C were significantly larger in the hyper-insulinemic group compared with the control or normo-insulinemic groups. Hyperinsulinemia and/or insulin resistance may largely contribute to postprandial increase in remnant lipoproteins in patients with type 2 diabetes.

Postprandial hyperlipidemia is closely related to postprandial hyperglycemia, and insulin resistance may be an enhancing factor for both postprandial hyperlipidemia and postprandial hyperglycemia. 

### 4.4. Differences in the Management of Postprandial Hyperglycemia and Postprandial Hyperlipidemia

IGT is defined by two-hour 75 g oral glucose tolerance test values of 140 to 199 mg/dL; normal values on this test are below 140 mg/dL [87]. Therefore, postprandial hyperglycemia can be considered to be more than 140 mg/dL. IGT is considered to be a prediabetic condition that will develop into diabetes in the future. Recently, a number of RCTs have been conducted in patients with IGT to study whether interventions using lifestyle modification [88,89] and anti-diabetic drugs such as acarbose, metformin, and pioglitazone [89,90,91] can delay the progression to diabetes. Such interventional studies for IGT have succeeded in delaying the development of diabetes. 

A fasting serum TG of 150 mg/dL or higher and a non-fasting serum TG of 175 mg/dL or higher have been adopted as diagnostic criteria for dyslipidemia in Japan [22]. The target value for non-fasting TG is less than 175 mg/dL, to prevent the development of ASCVD. However, postprandial hyperlipidemia is not specifically defined by such TG levels 2 h after a meal. A clear numerical target is needed to study interventions for postprandial hyperlipidemia to prevent the development of ASCVD. Furthermore, unlike plasma glucose levels, high TG levels do not necessarily cause atherosclerosis, because high TG levels due to elevated CM are unlikely to develop into atherosclerosis. These issues make the management of postprandial hyperlipidemia difficult.

## 5. The Atherogenic Properties of Remnant Lipoproteins and Metabolic Disorders Associated with Postprandial Hyperlipidemia 

**Figure 5:**
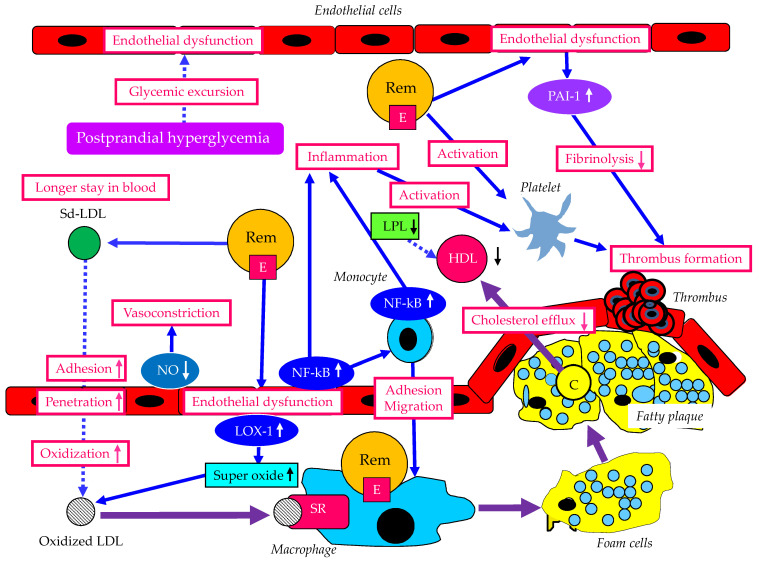
The atherogenic properties of remnant lipoproteins and metabolic disorders associated with postprandial hyperlipidemia. Solid purple bold lines indicate the flow of substances and cells. Solid blue lines indicate the abnormal effects induced by remnant lipoproteins. Dotted blue lines indicate the abnormal effects induced by metabolic disorders associated with postprandial hyperlipidemia. Red, white and black upward and downward arrows indicate an increase or a decrease in each factor, respectively. C, cholesterol; E, apo E; HDL, high-density lipoprotein; LDL, low-density lipoprotein; LOX-1, lectin-like oxidized LDL receptor-1; LPL, lipoprotein lipase; NO, nitric oxide; PAI-1, plasminogen activator inhibitor-1; Rem, remnant lipoproteins; Sd-LDL, small dense LDL; SR, scavenger receptor.

The atherogenic properties of remnant lipoproteins and metabolic disorders associated with postprandial hyperlipidemia are shown in Figure 5. Compared with CM or VLDL, CMR and VLDLR become enriched in apo E [13]. While CM and VLDL are prohibited from transcytosis by virtue of their size, remnant lipoproteins can penetrate the artery wall. Differing from LDL, which is deficient in apo E and requires oxidation for uptake, remnant lipoproteins do not need oxidation to facilitate accumulation in macrophages due to their apo E enrichment. Remnant lipoproteins increase the rolling and adhesion of monocytes on endothelial cells and transmigration between endothelial cells. Remnant lipoproteins are implicated in inflammation, platelet activation and endothelial dysfunction, by activating transcription factors such as NF-κB in endothelial cells and monocytes. In dysfunctional endothelial cells, plasminogen activator inhibitor-1 (PAI-1) expression increases, which reduces fibrinolysis, resulting in prothrombotic state. Reduced Nitric oxide (NO) in endothelial cells induces vasoconstriction. Remnant lipoproteins stimulate NAD(P)H oxidase-dependent superoxide formation in endothelial cells via the activation of the lectin-like oxidized LDL receptor-1 (LOX-1). Such oxidative stress increases the production of oxidized LDL, and oxidized LDL induces the formation of foam cells via scavenger receptors.

In the pathological condition that develops postprandial hyperlipidemia, HDL decreases due to decreased LPL activity, resulting in reduced cholesterol efflux from atherosclerotic plaque. High levels of remnant lipoproteins are likely to be accompanied by increased Sd-LDL. The increased atherogenic potential of Sd-LDL is suggested by its greater propensity for transport into the subendothelial space, increased binding to arterial proteoglycans, and susceptibility to oxidative modification [13]. Sd-LDL is cleared more slowly from plasma than LDL because LDL-R cannot recognize Sd-LDL.

Postprandial hyperlipidemia is likely to be complicated by postprandial hyperglycemia, and glycemic excursion observed in postprandial hyperglycemia induces endothelial dysfunction [92,93,94].

## 6. Treatments for Postprandial Hyperglycemia

### 6.1. The Modification of Lifestyle, Including Diet and Exercise

A diet high in minimally processed, high-fiber, plant-based foods such as vegetables and fruits, whole grains, legumes, and nuts will markedly improve postprandial hyperlipidemia [95]. Lean protein, vinegar, fish oil, tea, cinnamon, calorie restriction, weight loss, exercise, and low-to-moderate-dose alcohol consumption positively impact postprandial hyperlipidemia [95]. 

### 6.2. Anti-Hyperlipidemic Drugs

#### 6.2.1. Pemafibrate

Pemafibrate is a selective peroxisome proliferator-activated receptor alpha (PPARα) modulator, and a promising drug for postprandial hyperlipidemia. A meal tolerance test demonstrated that the AUCs of postprandial TG, apo B48, and RemL-C levels significantly decreased after the pemafibrate treatment [96]. The iAUC for postprandial TG levels was significantly reduced following the pemafibrate treatment (−39.7 ± 71.2%, *p* = 0.004). The peaks in the postprandial TG, apoB-48, and RemL-C levels after pemafibrate treatment occurred earlier than those at baseline and after placebo treatment [96]. Our previous study showed that pemafibrate significantly reduced both non-fasting TG and non-HDL-C levels at 3, 6 and 12 months after the start of pemafibrate [97], suggesting a possible beneficial effect of pemafibrate on postprandial hyperlipidemia.

#### 6.2.2. Fenofibrate

Fenofibrate, a PPARα agonist, reduced the integrated postprandial TG concentrations corrected for the fasting TG level, and postprandial CM concentrations, as assessed via biosynthetic labeling of CM with retinyl palmitate, 40.6% (*p* < 0.05) and 60.1% (*p* < 0.05), respectively, in patients with type IIB hyperlipoproteinemia [98]. In this study, fenofibrate increased LPL activity by 33.6% (*p* < 0.05).

The OFLT showed that fenofibrate reduced postprandial TG, TRL-TG, TRL-C, TRL-apo CIII, and TRL-apo E levels by −35% (all values of *p* < 0.01) in hypertriglyceridemic men with low HDL-C [99]. In the OFLT, fenofibrate showed a reduction in postprandial CM and VLDL concentrations as compared with placebo, in patients with type 2 diabetes [100].

Fenofibrate lowered postprandial TG-AUC (−45.4%, *p* < 0.0001) due to significant reductions in postprandial levels of large VLDL (−40.8%, *p* < 0.0001) and medium VLDL (−49.5%, *p* < 0.0001). Fenofibrate also reduced Sd-LDL by −40.3% (*p* < 0.0001) [101]. Fasting and postprandial oxidized FA were reduced by fenofibrate compared with placebo (−15.3%, *p* = 0.0013, and 31.0%, *p* < 0.0001, respectively), and fenofibrate lowered fasting and postprandial levels of soluble vascular cell adhesion molecule-1 (VCAM-1) and intercellular adhesion molecule-1 (ICAM-1). Reductions in VCAM-1 and ICAM-1 were correlated with reductions in fasting and postprandial large VLDL particles (*p* < 0.0001) and postprandial oxidized FA (*p* < 0.0005).

#### 6.2.3. Bezafibrate

The cookie test showed that bezafibrate, a PPARα agonist, significantly suppressed postprandial elevation of TG (iAUC: 544 ± 65 vs. 1158 ± 283 mg h/dL, *p* = 0.02) and remnant lipoprotein cholesterol (iAUC: 27.9 ± 3.5 vs. 72.3 ± 14.1 mg h/dL, *p* < 0.01) [102]. The postprandial TG content of CM and VLDL was significantly lower in the bezafibrate group than in the control group (*p* < 0.05) [102].

#### 6.2.4. Ezetimibe

The effects of ezetimibe on postprandial hyperlipidemia and hyperinsulinemia were assessed in obese metabolic syndrome patients with CAD by using OFLT. Serum TG and insulin levels (2 h after the loading dose) were significantly higher in metabolic syndrome patients than in control patients [103]. The iAUCs for TG and insulin levels decreased significantly after ezetimibe treatment in metabolic syndrome patients. When metabolic syndrome patients were divided into two groups based on the median insulin iAUC reduction rate (higher group ≥ 34%, *n* = 14; lower group < 34%, *n* = 13), those in the higher group showed a significantly higher rate of change in the iAUCs of TG than those in the lower group (TG, 31.0% vs. 10.8%; *p* = 0.033). These results suggest that ezetimibe may reverse insulin resistance, reducing lipid dysmetabolism after a meal in metabolic syndrome patients with CAD.

#### 6.2.5. Eicosapentaenoic Acid (EPA)

The cookie meal test showed that the EPA n-3 FA significantly decreased the incremental TG peak, the AUC for postprandial TG, the incremental glucose peak, and the AUC for postprandial glucose [104].

### 6.3. Anti-Diabetic Drugs

#### 6.3.1. Metformin

In nine glucose-intolerant subjects, metformin reduced the postprandial insulin AUC after OFLT from 389 to 245 mU/mL (*p* < 0.01), and also reduced CM and non cm retinyl palmitate AUC by 56% and 32%, respectively [105].

#### 6.3.2. Alpha-Glucosidase Inhibitors

Acarbose inhibited the postprandial increase in both plasma glucose and serum insulin [106]. Acarbose also significantly suppressed the increase in serum TG at 60, 90, and 120 min (*p* < 0.05 to *p* < 0.01), and the increase in serum RLP-C at 60 and 120 min (*p* < 0.05). Acarbose inhibited the postprandial decline in apolipoprotein CII, and decreased the level of postprandial serum apolipoprotein CIII. Alpha-glucosidase inhibitors may improve postprandial hyperlipidemia as well as postprandial hyperglycemia in patients with type 2 diabetes.

#### 6.3.3. Pioglitazone

Insulin resistance and both fasting and postprandial TG and RLP-TG levels decreased significantly after administration of pioglitazone in insulin-resistant smokers [107]. The postprandial accumulation of RLP-C is increased in insulin-resistant smokers and is likely to contribute to the increased CVD risk in such individuals. Pioglitazone administration provides a possible therapeutic approach to decreasing postprandial hyperlipidemia and CVD risk in insulin-resistant smokers.

The effects of pioglitazone and glibenclamide (sulfonylurea) on insulin resistance, postprandial hyperglycemia, and postprandial hyperlipidemia were examined in type 2 diabetic patients [108]. Pioglitazone significantly reduced fasting plasma glucose (FPG), HbA1c, the homeostasis model assessment of insulin resistance (HOMA-IR), and TG compared to baseline. In contrast, glibenclamide significantly reduced FPG and HbA1c, while not affecting HOMA-IR and TG. Pioglitazone significantly improved postprandial TG and RLP-TG; however, glibenclamide did not show an influence on postprandial TG and RLP-TG. 

#### 6.3.4. Dipeptidyl-Peptidase-4 Inhibitors (DPP4is)

DPP4is have been demonstrated to improve glycemic control, in particular postprandial hyperglycemic control, in patients with type 2 diabetes. A standard meal loading test showed that alogliptin treatment significantly suppressed the postprandial elevation in serum TG (iAUC; 279 ± 31 vs. 182 ± 32 mg h/dL, *p* = 0.01), apoB-48 (iAUC; 15.4 ± 1.7 vs. 11.7 ± 1.1 μg h/mL, *p* = 0.04), and RLP-C (iAUC: 29.3 ± 3.2 vs. 17.6 ± 3.3 mg h/dL, *p* = 0.01) in non-diabetic subjects [109]. Vildagliptin showed a better effect on postprandial hyperlipidemia and hyperinsulinemia compared to glimepiride in patients with type 2 diabetes [110].

#### 6.3.5. Glucagon-like Peptide 1 Analogues (GLP1A)

Liraglutide reduced apo B48 synthesis in CM by 60% (*p* < 0.0001), as well as VLDL-TG secretion (*p* = 0.017), in parallel with reduced liver fat in patients with type 2 diabetes [111]. CM-apo B48 production was related to insulin sensitivity (*p* = 0.015). A standardized fat-rich meal load test showed that liraglutide significantly reduced postprandial TG and apo B48 AUC, more than placebo, by 28% and 35%, respectively [112]. Liraglutide treatment significantly reduced postprandial excursions of TG and apo B48 after a fat-rich meal, independent of gastric emptying, in patients with type 2 diabetes. Exenatide significantly reduced postprandial glucose, TG, apo B48, VLDL-C, FFA and the marker for oxidative stress, malondialdehyde (MDA) excursions, whereas insulin glargine did not show an influence on postprandial TG, apo B48 and VLDL-C [113]. Changes in oxidative stress makers were significantly associated with changes in postprandial glucose and TG excursions, independent of the treatment arm. Our previous studies showed that subcutaneous dulaglutide and oral semaglutide reduced non-HDL-C [114,115], supporting a beneficial effect of GLP-1A on postprandial hyperlipidemia.

#### 6.3.6. Sodium Glucose Cotransporter-2 Inhibitors (SGLT2is)

The 8 week-tofogliflozin (SGLT2is) treatment caused an improvement in postprandial glucose metabolism; however, it did not show an improvement in considerable postprandial lipid metabolism in Japanese men with type 2 diabetes [116]. Although the OFLT showed that the 12 week-dapagliflozin treatment did not affect fasting or postprandial plasma cholesterol and TG, fasting apo B48 was decreased by dapagliflozin [117]. Empagliflozin decreased RLP-C and HOMA-IR in type 2 diabetic patients with insulin resistance [118]. The change in RLP-C was significantly correlated with the change in HOMA-IR (Pearson’s correlation coefficient, 0.503; 95%CI, 0.199 to 0.719; *p* = 0.00241). Our previous study showed that SGLT2i significantly reduced non-HDL-C at 3 months after the start of SGLT2i, suggesting a promising therapeutic effect to counter postprandial hyperlipidemia [119,120]. Reduced levels of fasting apo B48, RLP-C, and non-HDL-C caused by SGLT2is suggest a possible beneficial effect of SGLT2i on postprandial hyperlipidemia.

## 7. The Limitations of the Strengths of Our Review Article

Our main limitation is the small percentage of references from the last 5 years, with a significant share of references being over 10 years old. We have described the pathophysiology of the well-known links between postprandial hyperlipidemia and post-prandial hyperglycemia, postprandial FFA and TRL, on the basis the references that are over 20 years old, which may be not very scientific and innovative. Our limitations are due to the recent lack of high-evidence-level papers on postprandial hyperlipidemia, and also due to the lack of established guidelines for postprandial hyperlipidemia.

There is no comprehensive review on postprandial hyperlipidemia including its pathophysiology, diagnosis, atherogenesis and treatments. We believe that our review article will be useful for promoting comprehensive understanding of postprandial hyper lipidemia, which is the strength of our article.

## 8. Conclusions

Postprandial hyperlipidemia is atherogenic and associated with the development of CAD. It is important to appropriately understand the existence of postprandial hyperlipidemia and to connect it to optimal treatments. However, there are some problems with the diagnosis of postprandial hyperlipidemia. Elevations in fasting and non-fasting TG, non-HDL-C, fasting serum apo B48, RLP-C and Rem-C were useful to identify the existence of postprandial hyperlipidemia; however, since reference values for these parameters have not been determined, no treatment strategy has been established based on these values. Postprandial hyperlipidemia cannot specifically be defined by measures such as TG levels 2 h after a meal. To study interventions for postprandial hyperlipidemia with the outcome of preventing the onset of ASCVD, it is necessary to define postprandial hyperlipidemia using reference values such as IGT. 

## Data Availability

Not applicable.
